# 

**Published:** 1907-03

**Authors:** 


PUBLISHED MONTHLY.

### ATLANTA JOURNAL-RECORD OF MEDICINE.

Successor to Atlanta Medical and Surgical Journal, Established 1855. and Southern Medical Record, Established 1870.

Vol. VIII. ATLANTA, GA., MARCH, 1907. No. 12.

